# Adapting an Evidence-Based e-Learning Cognitive Behavioral Therapy Program Into a Mobile App for People Experiencing Gambling-Related Problems: Formative Study

**DOI:** 10.2196/32940

**Published:** 2022-03-25

**Authors:** Gayl Humphrey, Joanna Ting Chu, Rebecca Ruwhiu-Collins, Stephanie Erick-Peleti, Nicki Dowling, Stephanie Merkouris, David Newcombe, Simone Rodda, Elsie Ho, Vili Nosa, Varsha Parag, Christopher Bullen

**Affiliations:** 1 National Institute for Health Innovation University of Auckland Auckland New Zealand; 2 Hapai Te Hauora Auckland New Zealand; 3 Deakin University Melbourne Australia; 4 School of Psychology Deakin University Melbourne Australia; 5 Social and Community Health University of Auckland Auckland New Zealand; 6 Pacific Health University of Auckland Auckland New Zealand

**Keywords:** gambling, CBT, mHealth, co-design, smartphone, self-directed, behavior change, engagement, mobile phone

## Abstract

**Background:**

Many people who experience harm and problems from gambling do not seek treatment from gambling treatment services because of personal and resource barriers. Mobile health (mHealth) interventions are widely used across diverse health care areas and populations. However, there are few in the gambling harm field, despite their potential as an additional modality for delivering treatment and support.

**Objective:**

This study aims to understand the needs, preferences, and priorities of people experiencing gambling harms and who are potential end users of a cognitive behavioral therapy mHealth intervention to inform design, features, and functions.

**Methods:**

Drawing on a mixed methods approach, we used creators and domain experts to review the GAMBLINGLESS web-based program and convert it into an mHealth prototype. Each module was reviewed against the original evidence base to maintain its intended fidelity and conceptual integrity. Early wireframes, design ideas (look, feel, and function), and content examples were developed to initiate discussions with end users. Using a cocreation process with a young adult, a Māori, and a Pasifika peoples group, all with experiences of problem or harmful gambling, we undertook 6 focus groups: 2 cycles per group. In each focus group, participants identified preferences, features, and functions for inclusion in the final design and content of the mHealth intervention.

**Results:**

Over 3 months, the GAMBLINGLESS web-based intervention was reviewed and remapped from 4 modules to 6. This revised program is based on the principles underpinning the transtheoretical model, in which it is recognized that some end users will be more ready to change than others. Change is a process that unfolds over time, and a nonlinear progression is common. Different intervention pathways were identified to reflect the end users’ stage of change. In all, 2 cycles of focus groups were then conducted, with 30 unique participants (13 Māori, 9 Pasifika, and 8 young adults) in the first session and 18 participants (7 Māori, 6 Pasifika, and 5 young adults) in the second session. Prototype examples demonstrably reflected the focus group discussions and ideas, and the features, functions, and designs of the Manaaki app were finalized. Attributes such as personalization, cultural relevance, and positive framing were identified as the key. Congruence of the final app attributes with the conceptual frameworks of the original program was also confirmed.

**Conclusions:**

Those who experience gambling harms may not seek help. Developing and demonstrating the effectiveness of new modalities to provide treatment and support are required. mHealth has the potential to deliver interventions directly to the end user. Weaving the underpinning theory and existing evidence of effective treatment with end-user input into the design and development of mHealth interventions does not guarantee success. However, it provides a foundation for framing the intervention’s mechanism, context, and content, and arguably provides a greater chance of demonstrating effectiveness.

## Introduction

### Background

Gambling problems are viewed across a continuum of risk in New Zealand, ranging from no risk to problem gambling, where high-risk gambling behavior results in considerable health and social problems. Consistent with this framework, the term problem gambling is described as any kind of harm, distress, or adverse impact caused or exacerbated by a person’s gambling [1-3]. Gambling-related harm is a significant public health problem in New Zealand [4,5] and internationally [6-8]. In New Zealand, the latest prevalence estimates suggest that 0.1% of the population experiences problem gambling, with a further 1.5% and 3.3% experiencing moderate- and low-risk gambling, respectively [9].

Different treatment service modalities, such as face-to-face counseling, phone support, self-management workbooks, and web-based tools, such as those provided on the Safer Gambling Aotearoa website [10], are readily available, although none are mobile health (mHealth) apps. A recent survey reported that only 3.2% of people with problem gambling had accessed at least one type of support service [9]. Therefore, a significant proportion of the population does not receive optimal therapeutic support [11]. In New Zealand, the Māori (indigenous people of New Zealand) and Pasifika peoples are disproportionately impacted by problem gambling [12,13]. Although some theories of addiction help explain gambling addiction attributes [14], the influence of historical and cultural intergenerational experiences is equally important in understanding the gambling context for Māori [15,16]. Thus, it is vital that interventions accommodate and reflect the cultural appropriateness and presentation of content relevant to their needs and understanding.

The ubiquity of smartphone ownership and the exponential growth of new mobile technology functionality provide an opportunity to add new modalities for delivering evidence-based health interventions [17]. mHealth apps, defined as the use of mobile and wireless technologies for health promotion [18], have extended support for health behavior changes beyond standard treatment contexts. These interventions continue to be in demand, with a reported 3.7 billion mHealth downloads in 2017 [19] and an expected 29% growth rate over 2020-2027, equating to a forecast of 311.9 billion by 2027 [20]. Nonetheless, the retention and use of mobile apps is less than ideal, with reports suggesting that most are uninstalled within 5 days, with mHealth apps deleted slightly later, at 8.8 days [21]. However, app reinstalls are also not unusual, with mHealth apps reporting a reinstall rate of 14% [21].

Some mHealth research reviews explain that the lack of underpinning theory to support the app’s intent (ie, the set of determinants that influence cognitive and behavior change assertions) contributes to the lack of app retention and hence the paucity of demonstrable evidence of effectiveness [22-27]. Other mHealth research highlights that the lack of involvement of intended end users or consumers in the design and development is an essential yet missing ingredient in creating effective mHealth interventions [28,29].

Therefore, informing mHealth interventions with research evidence, insights from domain experts, and a theoretical framework for high-fidelity interventions are critical [30], as is the involvement of consumers and intended end users [31-34]. Blending these strategies into concrete elements for digital interventions is not straightforward [35]. Although adopting this approach does not guarantee success [36,37], it can optimize the creation of an effective mHealth program. To that end, there has been a call to publish research that outlines the development processes underpinning mHealth interventions [38,39]. Publishing and sharing such research, which involves both mapping theoretical constructs and cocreation with end users, is a positive step in building a robust and replicable evidence base.

### Objectives

In this study, we describe the formative research that was an integral part of developing a mobile app for gambling harm for use in New Zealand: Manaaki. Specifically, we aim to (1) adapt and refine an evidence- and web-based program, GamblingLess [40-42], into a mobile app intervention and (2) engage domain experts and intended end users to help inform the content conversion and the design, features, and functions of the app and maximize the effectiveness of the program. This study is part of a larger project to evaluate the effectiveness of this designed cognitive behavioral therapy (CBT) mHealth intervention—Manaaki—for people with self-reported gambling problems [43].

## Methods

### Ethics Approval

Ethical approval was obtained from the New Zealand Health and Disability Ethics Committee (19/STH/100). As part of the ethics consenting process**,** participants gave consent for a summary of the focus group contributions, including quotes where relevant, to be included in publications as part of the participant consenting process.

### Development Process

The development process encompassed 2 key phases.

Phase 1 consisted of the research team reviewing the current GamblingLess program and adapting and refining it into a first draft prototype of content, features, and functions suitable for a mobile phone–delivered intervention.

Phase 2 involved a qualitative approach using repeated focus groups and prototype testing to engage end users as collaborative design partners. This approach was used to inform and refine the app’s features, style and functions, and content presentation.

### Phase 1: Intervention Content Development

#### Overview

The multidisciplinary research team included clinical gambling research domain experts, including GamblingLess [40] program creators (Associate Prof Dowling, Dr Merkouris, and Dr Rodda, Deakin University, Australia); mHealth and digital health experts; public health physicians; Māori, Pacific, and Asian health addiction research experts; a statistician; and 2 researchers from Hāpai te Hauora (an Iwi-led [groupings of the indigenous population based on kinship] community health organization). Collectively, these team members provided expert advice to unpack the program’s core elements to ensure that the content remained evidence based. The Aotearoa New Zealand context also informed practical and clinical considerations during the design and development phases.

#### Unpacking GamblingLess

GamblingLess [42] is a comprehensive and intensive web-based CBT program designed to help people with gambling problems. It was designed to emulate the intensity and depth of the face-to-face programs. The original program content included 4 modules that were delivered over 8 weeks. The modules included motivational enhancement, behavioral strategies, cognitive strategies, and relapse prevention, and each module included 13-15 discrete activities. Although it was recommended that participants complete all modules and activities in numerical order, they were allowed to complete as many activities as they liked, in any order they chose.

Associate Prof Dowling, with the support of Dr Merkouris and Dr Rodda, initially reviewed the 4 modules of GamblingLess, reflecting on the findings from their original research [41]. This revised program intentionally maintained the original GamblingLess principles underpinned by the transtheoretical model [44]. The key was to acknowledge that some end users (help seekers) would be more ready to change than others. As change is a process that unfolds over time, a nonlinear progression is needed, enabling the end users to select the interventions they need according to their stage in the change process.

Accordingly, the goal of this redeveloped program is to support the end user in selecting and using the most appropriate module or modules based on their needs. It is designed to be nonlinear, and hence presents the end user with a range of options (modules) with which one or more resonates with what they hope to achieve; that is, managing urges or relapse prevention. Therefore, it was anticipated that not all end users would need or want all modules or all activities within the program.

To create Manaaki, the acceptability data from the original GamblingLess program were also reviewed, with the most helpful treatment content identified and assessed for inclusion in this program [41]. The outcome of this revision was that the original 4 treatment modules were expanded to 6 modules. The research team reviewed the 6 new modules and provided initial suggestions on how each module and associated actions or activities might be adapted to fit a smartphone mobile app interface. Iterative cycles of face-to-face meetings, web-based meetings, and email conversations were used to create and refine the core structure, content, and activities of the 6 new modules.

The researchers adopted the consumer engagement strategies common in marketing [45,46], and with a user experience designer and research partners from Hāpai te Hauora, ideas and examples for the draft elements for each module and the control app were drafted. These ideas were translated into the following mixed medium modalities for use during the cocreation phases with end users:

- A draft wireframe to illustrate flow and give depth and perspective using Adobe XD (Adobe Inc)
- Large A0-sized posters of each module’s wireframes, including color palette ideas
- A set of A4-sized paper mobile phone screens to show draft content
- A set of various images for app screens

### Phase 2: Cocreation With End Users

#### Overview

We used a qualitative cocreation methodology [47] to determine the look, feel, function, and features of the Manaaki intervention app. As gambling impacts more marginalized and economically deprived populations, the research team created three groups: a Māori group, a Pasifika group, and a specific young adult group that was limited to participants aged 18-25 years. These groups were selected to maximize the app’s acceptability for those most likely to benefit the most. In all, 3 cocreation cycles were planned for each group. This approach encourages increased involvement and ownership of the process by participants, as they can see how their contributions help shape each design iteration of the app [48].

Researchers from Hāpai te Hauora led participant recruitment. Through their networks, contact was made with various community groups and organizations to help identify potential participants for the different groups. The eligibility criteria were as follows: (1) having experienced problems or harms from their gambling or supporting someone who is or has experienced problems with gambling, (2) able to understand and converse in English, (3) able to participate in at least two of the three cocreation cycles, and (4) aged ≥18 years for the Māori and Pacifika groups or 18-25 years for the young adult group.

The focus groups were conducted between July and November 2019. The focus groups for the Māori and Pasifika groups were held in Auckland, whereas for the young adult group, it was held in Wellington. Each workshop lasted between 60 and 90 minutes and was audio recorded with notes taken. The workshops were facilitated by the Hāpai te Hauora researchers and were attended by the design experts.

A mixture of methods was used to elicit feedback and comments from participants. These included open-ended questions, iteratively adding to or removing elements on the Adobe XD wireframes, Post-it notes, writing on A0-sized posters, and ad hoc prompts for a more detailed discussion when new ideas emerged.

After each focus group, various engagement modalities (posters and Adobe XD wireframes) were adapted to reflect emerging ideas and discussions. These were incorporated into the next prototype version, ready for participants to reassess, discuss, and modify the subsequent focus group sessions.

As an appreciation of their time, each participant received an NZD $30 (US $20.30) voucher to attend the first focus group and an NZD $20 (US $ 13.50) voucher for each additional focus group attended.

#### Data Analysis

The data for the focus groups were analyzed using a general inductive approach [49]. The purpose of using an inductive approach was to (1) condense raw textual data into a brief summary format (using images, words, wireframes, and prototypes), (2) establish clear links between the research objectives and the summary findings derived from the raw data, and (3) develop a framework of the underlying structure of experiences or processes that are evident in the raw data. All focus group data were grouped under the relevant themes, unless it was pertinent to describe a particular group’s findings separately. All direct quotes are in *italics* and illustrate specific conversation points.

## Results

### Phase 1: Converting the Internet-Based GamblingLess Program to a Draft mHealth Program

**Figure 1:**
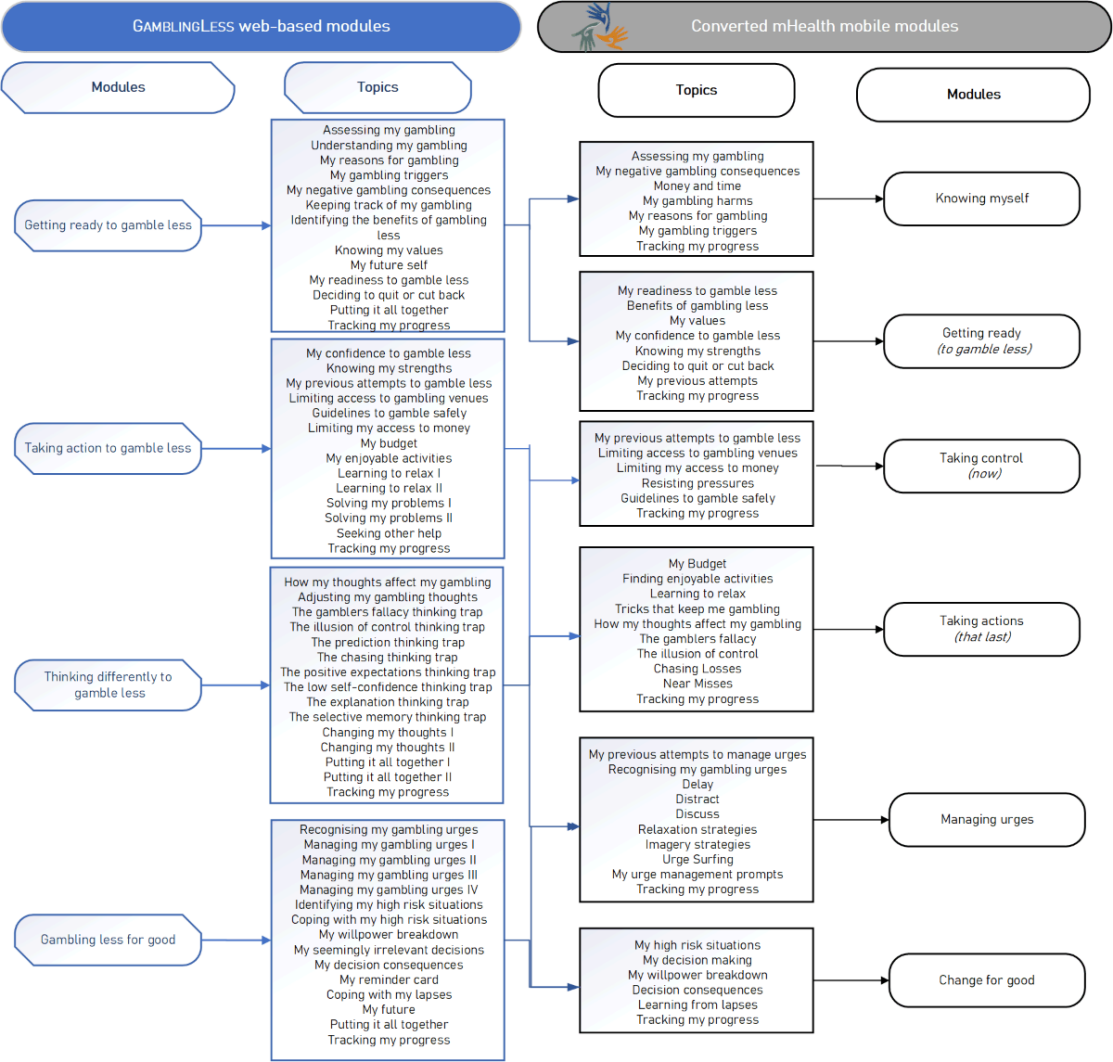
Mapping web-based GAMBLINGLESS program content to the Manaaki mobile app content. mHealth: mobile health.

On the basis of the GamblingLess research findings, the original 4 core modules were altered into 6 new modules, each with 5-7 learning topics. Each learning topic had a set of activities or interactions to support the learning intent of the module’s theme (Figure 1).

Each new module was carefully reviewed and remapped to the underpinning conceptual frameworks and intent to ensure fidelity and conceptual integrity (Table 1).

The outcome of this process enabled early wireframes, design ideas (look, feel, and function), and content examples to be drafted, which helped initiate discussions and ideas with end users during phase 2 and cocreation with end users.

**Table 1:** Remapping modules to concepts and the intervention intent.

Module names	Conceptual framework	Underpinning intent
Knowing Myself (and my gambling)	Developing self-awareness and insight [50-52]	Designed to provide personalized feedback, goal setting, and understanding of gambling motivations, triggers, and consequences.
Getting Ready (to make changes)	Targeting thoughts and feelings and activating behaviors [53]	Designed to enhance readiness and confidence to gamble less, helping to shape thoughts and values to help make a change. Incorporates ideas of prediction of outcomes from their behaviors.
Taking Control (right now)	Targeting practical behaviors and identification of situational and contextual triggers	Designed to identify strategies to be used to contain and control the problematic gambling behavior in the short term. Directs the end user to other useful tools, such as venue exclusions, managing money, services and other resources.
Taking Actions (that last)	Activating personal strengths and resources and enhancing belief for successful change [54,55]	Designed to identify strategies and skills to be used to ensure longer-term success in gambling less.
Managing Urges (to cope with real situations)	Reframing thoughts and reflecting on your future self [54,55] and relapse prevention [56] (see also Marlatt and Gordon, 1985)	Designed to help the end user to cope with urges and cravings.
Change for Good (and building a new future)	Relapse prevention [56]	Designed to prevent gambling relapses in the future and to support cognitive capability to understand their lived experience of gambling harms and problems as a mechanism for maintaining their new behavior.

### Phase 2: Cocreating and Designing With End Users

#### Overview

**Figure 2:**
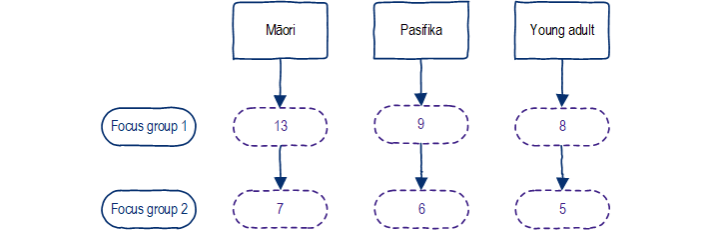
Focus groups and participant numbers. FG: focus group.

In all, 2 rounds of focus groups were conducted, with 30 unique participants (13 Māori, 9 Pasifika, and 8 young adults) in the first session and 18 participants (7 Māori, 6 Pasifika, and 5 young adults) in the second session (Figure 2). The time between the 2 focus group sessions varied from 4 to 7 weeks among the groups, as arranging an agreeable time for all participants was difficult.

#### Focus Group Cycle 1

##### Gathering Experiences

Participants were encouraged to think aloud and share difficulties when they or a loved one experienced gambling problems. The concept of *whakama* or shame was the dominant theme across all groups. Some participants remarked that they did not want to acknowledge that they had a problem with their gambling and had a strong desire to keep their gambling problems a secret. Consequently, few reported seeking professional help, but most remarked that they had completed a web-based quiz that assessed their gambling. Even when the quiz results suggested that they may have had a significant gambling problem, taking action was self-reported as trying the strategies that the websites had recommended. Although most remarked that these strategies were helpful, many found that they had returned to their previous harmful gambling behavior. A participant remarked that they felt that without support or reminders, it was easy to forget the learnings. The idea of a mobile phone intervention (*app*) that could provide help wherever and whenever needed was positively established across all 3 groups.

Initially, all groups discussed and conceptualized the app as a one-off tool to be used and then discarded it. However, as the participants continued to share their experiences and discuss what they would find helpful, the concept of a one-off tool was abandoned. The app was increasingly described as something that would be used to *help now and later.* Terms such as *faigamalaga* (Pacific group) or *haerenga* (Māori group) were used to convey the idea of recovery and about being on a journey or voyage. The Māori focus group also discussed the concept of *kotahitanga* (togetherness and sharing) as an important concept and that recovery with whānau (family or extended family) is crucial, as it is “through our whānau āwhina” (extended family support) “that we can be and stay well.”

##### The Look and Feel of the Intervention App

In all, 12 draft design images were presented to the participants, and it was clear that the participants expressed excitement in seeing their ideas of a journey already coming to life. As each focus group began selecting images that they liked, conversations within the groups became energetic. Although all 3 groups navigated these discussions differently, their outcomes were similar. Each group concluded that keeping a range of different images of New Zealand was better than keeping a single image or theme.

**Figure 3:**
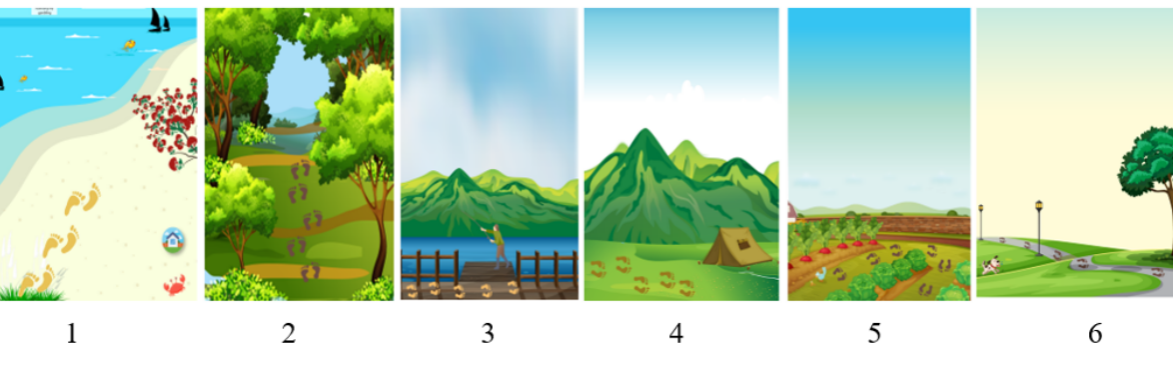
The 6 most popular initial concept imagery selected.

The 6 images that were the most popular across all 3 focus groups are presented in Figure 3.

In addition to selecting these images, engagement with the images was such that participants thought of ideas to enhance their look and feel, such as to “add more birds; have the bush image a bit more open” (Figure 3; image 2). The footprints were designed to illustrate movement; yet a Pasifika participant wanted to “remove the footprints,” whereas others in the group liked them. The footprints were not mentioned by the young adult or Māori group participants. The fisherperson and tent in Figure 3 (images 3 and 4, respectively) were disliked across all 3 groups, commenting that they “did not fit with the other images.” A few participants in the Māori focus group remarked that the perspective in these 2 images was different from the others, and “it didn’t work.” The young adult group remarked that Figure 3 (image 6) “needs people and needs some life in it. It's too sad looking, aye.” Similarly, the Pasifika group said Figure 3 (image 6) “looks a bit too lonely. Where is everyone?” The Māori group participants also commented that Figure 3 (image 6) needed “people and activity in that scene so that you felt part of the world, you know, just being normal with your whānau. Yeah, more life.”

##### Shaping the Features, Functions, and Content Desired

The conversations naturally segued from the look and appeal of the images to the important attributes (features and functions) needed to make the app useful and easy to use. The participants in all 3 focus groups began noting down the key features, functions, and some content elements they wanted in the app, using Post-it notes or writing directly on the posters, Textbox 1 summarizes these initial attributes.

**Early attributes to have in the app from the all first focus group sessions.**

**Features**

- Make it personal
- Able to share with others
- Progress and achievement
- Receive feedback
- Measuring where I am
- Self-checks
- Help guide me to where to go next
- Reminders and prompts
- Links to services if needed
- Tracking on how I am going
- Goals
- A place for reflection and celebration
- Quick access to other help if needed

**Functionality**

- Easy to use
- Navigation around makes sense
- No need to do everything
- Be interactive
- Short things to do
- Not too many clicks to find things
- Needs to be fun and have animation
- Quick, no lagging when loading

**Content**

- Simple language
- Use images
- Video
- Not too much text
- Positively framed
- Can listen not just read
- Self-help resources
- What other help is available
- Culturally appropriate
- Shows helpful skills
- Activities

**Cross-cutting elements**

- Secure, private, and confidential
- Easy to download, not complicated
- No own data use
- Not too intruding
- No cost
- Works offline

##### Creating Ownership of the App Name and Logo

In consultation with our Māori research team members, the name *Manaaki* was suggested as a possible name for the mHealth intervention app. The name Manaaki has a special meaning in Māori, which blends the words mana and āki. Mana means to bring strength or give strength and to cherish. Almost every activity is linked to the maintenance and enhancement of mana. The word āki encompasses the concept of encouragement or urge for support, as well as to hold fast and be strong. To ensure that this term was acceptable and appropriate, the team sought guidance from the governance group at Hāpai te Hauora (our Māori research partner organization). They supported the use of the word Manaaki as the name for the new app.

The focus group participants had positive remarks about the name Manaaki. “I love the name Manaaki; it is perfect,” said a Māori group participant. “It gives a positive vibe,” was reported by a young adult group participant, and a Pasifika participant remarked, “I like it. Manaaki is all about being encouraging, so perfect for this app.”

In all, 10 concept designs (images, colors, and text types) were created, and participants were encouraged to comment on the options provided and select the text, logo, and colors they liked or offered other ideas.

**Figure 4:**
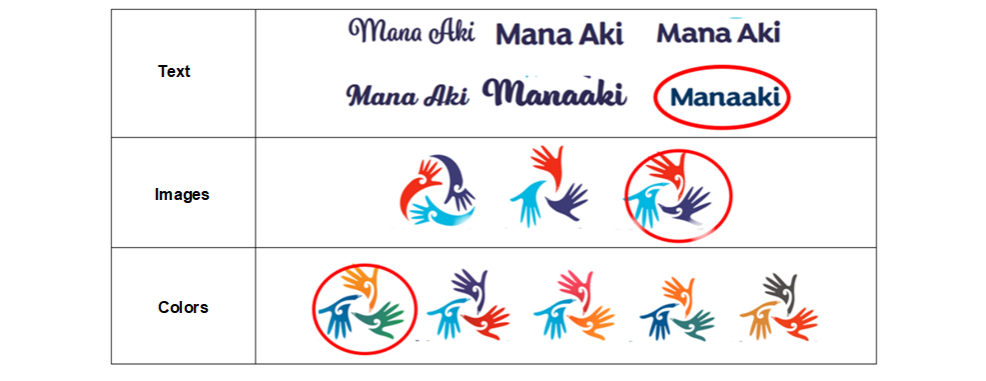
Concept designs for name and logo.

Figure 4 shows the text, images, and colors that were frequently selected, with those circled in red confirmed as the final designs.

##### Encouraging Recruitment Into the Study

The final discussion element for the first focus group was gathering insights into the study design—a randomized waitlist control study—and its impact on recruiting participants. It was evident that participants in all the groups understood that tools and treatments offered to people experiencing harm from gambling needed to be effective and that research is needed to prove effectiveness. Despite understanding the need to research the app for effectiveness, both the young adult group and the Pasifika group remarked, “that having to wait to get the full app is a put-off.” Similar remarks were made in the Māori group:

If you have to wait, you need to let us know that we can still go look for help. You know, that being in this study thing, doesn’t stop that. Gotta be real clear too, about the information saying that, all the stuff in the app will be able to be used, after the wait.

Māori focus group, P3

Although the idea of waiting was viewed as negative, having some functions was suggested as a way to help retention. The following comment clearly reflects this:

If I had made the decision to do something about my gambling, and then was told, oh wait for a bit. [Laugh] Well, I would think, that’s useless, and delete it and try something else. It may even put me off. But you know, seeing that there is still something for me, well maybe, [Laugh], that’s better than nothing. So I might stick with it a bit, who knows.

Young adult participant, P2

Functions and attributes commonly mentioned across the groups had a countdown icon showing how long to when the full Manaaki app would be available. The young adult group also suggested that the countdown icon be a clickable button that could *flip* and show the same outcome using different metrics, such as months, weeks, or days. Common to all groups was that the waitlist app needed to offer support within the app. Support such as links to services or websites was frequently noted, with the Pasifika group also mentioning that links to money management would also be good to have.

#### Focus Group Cycle 2

The purpose of the second focus group was to present the changes made to the designs and show the features and functions that were added or modified based on the group contributions from the focus group 1.

##### Look and Feel

**Figure 5:**
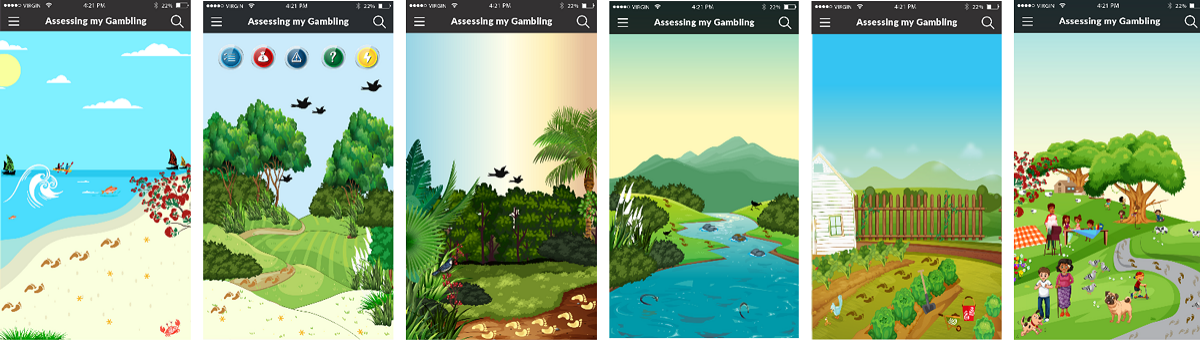
Modified screen imagery based on participant feedback.

The new image designs (Figure 5) and an animated version using Adobe XD were presented to each focus group, and they all received positive comments such as, “These are great. I love the colours, and the animation is sweet,” from a young adult participant.

**Figure 6:**
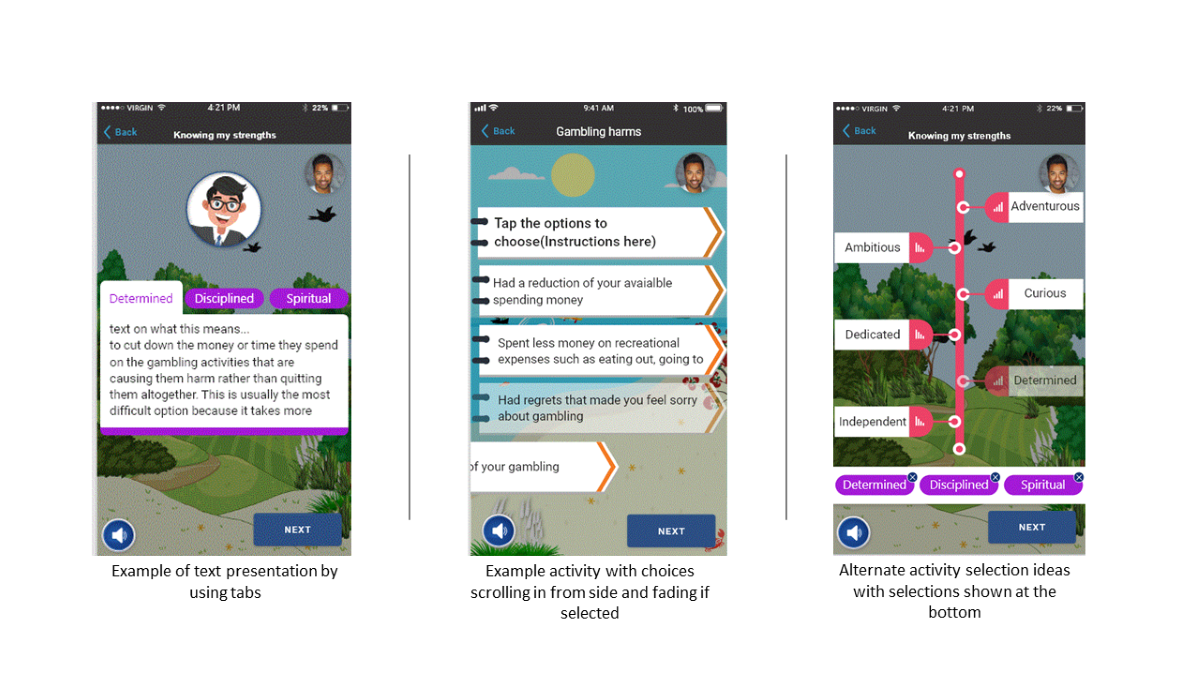
Example of ideas for presenting text and activity interactions.

All groups remarked that the various interactive content and features that support users engaging with and using the app were clear and intuitive. The use of different styles (images and themes) for different modules was received positively (Figure 6).

**Figure 7:**
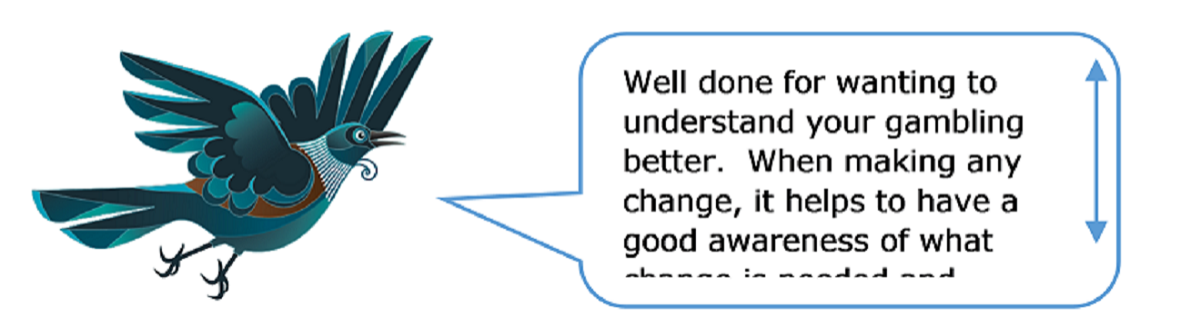
Tui guide.

An animated Tui (a native New Zealand bird [Figure 7]) was designed to appear at the beginning of each module to explain the learning intention and topics for the module. This information was presented in an auto-scrolling speech bubble next to Tui. The participants could also listen to the message using the audio option. This audio feature was highly liked and recommended by all the focus group participants, although a few comments from the Māori focus group remarked that the “computer voice is a bit annoying.” In contrast, some participants in the Pasifika focus group were excited by this option and thought this audio option could enable the text to be spoken in different languages.

Participants were noticeably excited when they saw how the text, images, and colors that were selected during their first focus group session formed the framework for the final branding of Manaaki.

##### Personalization

In focus group cycle 1, all participants expressed a strong desire for the app to be personalized. The examples that showed how a user’s first name would be integrated through the app were highly commended. Similarly, the use of language-specific greetings and phrases throughout the text was seen as acknowledging the population Manaaki intended to support. For many, these “little things [that will] help keep people engaged.” Similar comments were made when the function of uploading an image or selecting a personalized avatar to represent a user visually was presented as an idea for how the app could be personalized to the user.

Participants also acknowledged that people could be at different stages of their journey to reduce the behaviors contributing to their gambling-related harm. They all remarked that the designs reflected their ideas from the first focus group sessions, especially the way the app was designed to enable the user to navigate their journey and yet receive prompts and suggestions to help guide them. Other functions, such as the journal, the link to the helpline chat, and the services being visible using the maps, were remarked on as meeting different users’ needs and stages, making Manaaki useful to a diverse range of potential people.

##### Positivity

Positive framing and strength-based content were considered important, and the use of positive and simple language was repeatedly remarked on as being successfully shown across all focus groups:

I like how it is about knowing strengths.

Pasifika focus group, P6

Although some changes still such as the following needed to be made:

Some words are more researchy, like - Fallacy - wouldn’t be understood by most. Use a simple way to say this.

Māori focus group, P1

Maybe instead of looking at what are you spending, it could be, umm, learning to save?

Young adult focus group, P2

The range of notification message examples was presented as suggested in the first focus groups as an additional way to provide support and insights to a user as they progressed through activities in the app. These were positively remarked upon, and the young adult groups suggested that a mix of notifications and pop-ups would be more appealing than a single modality. The motivating and encouraging message examples “hit the spot,” said a young adult participant. All groups liked the notifications that prompted people to use the app, and many felt that this functionality would help with engagement and retention.

##### Ability to Share Insights and Progress With Others

The participants suggested that the app could share progress or success with others. This type of networking system was suggested as a positive method for users to encourage each other, share tips and strategies, and provide a sense of *not being alone*. This feature is technically complex, and as such, the solution presented to the groups was that sharing would be limited to those within the study. Feedback from all 3 groups indicated that they would have liked to connect with others beyond the study, as these broader social connections were important.

##### Tracking My Progress

Being able to track progress appealed to participants in focus group cycle 1. In total, 2 examples were presented as a way to represent progress. These were progress bars under the modules and the emergence of footprints on each module’s home screen. The progress bars were identified as the best across all groups, primarily because they were used in other apps and as such, were familiar and easy to understand.

In several instances, participants in the Pasifika group indicated that the app should be able to track the amount of money spent on gambling, money saved from not gambling, and how much time they had or had not spent gambling. Various ideas have been suggested for presenting this in the app. The design that resonated across all groups was one in which the time and money recorded as spent gambling was converted into other activities; for example, the number of dinners with family or friends or movies went to with friends.

##### Use Images and Multimedia

The app interface needed to be engaging and attractive to all the participants. The young adult group remarked that if the app looked unattractive at the start, they were unlikely to engage. Updated high-fidelity images were shown to the groups, and the following remark summarizes the generally positive comments made across all the groups:

Good background now that there is Pacific/Māori punch to it – waka, marae, native birds. Thanks.

Māori focus group, P11

Regarding the inclusion of multimedia, feedback was mixed, with participants in the Māori and Pasifika groups liking that short videos from real gamblers were part of the app. The young adults group participants were less enthusiastic, but they liked the cartoon animation videos than those with real people. Having different video styles was seen as a good option.

Overwhelmingly, all participants were concerned that having these videos in the app might affect the ability to download the app, that is, too large or that it would take up too much of their phone’s storage, slowing down their phone or stopping other functionality. Hearing that the videos would not be embedded within the app but would be stored externally and linked to YouTube generated positive remarks.

##### Connect With Treatment Services

**Figure 8:**
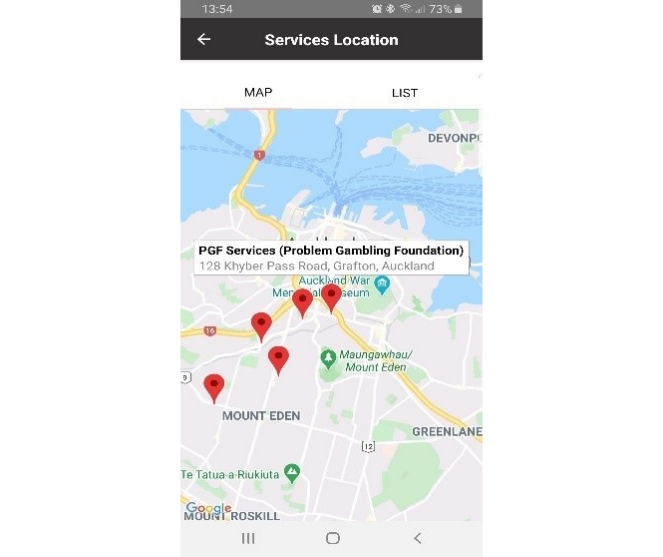
Image of the map search feature.

The participants in all groups agreed that connecting to a professional or treatment provider through the app would be advantageous. Participants were shown the services feature and interactive function that presented services by geography (map). List-based search options (Figure 8) were good options as illustrated by the following comments:

This feature is perfect, it allows users to look for help without needing to know the name or address of the place.

Pasifika focus group, P7

The app helps to find a service of your choice if you want to, it’s neat.

Young adult group, P1

The link to the gambling helpline chat function was highlighted. A participant in the Pasifika focus group commented that “this is like a rescue button. There if you need it*.”* Overall, the ability to access other help (not just the app) was reinforced as a core feature of Manaaki.

##### Work Offline

The ability of the app to be accessible offline and not require a Wi-Fi connection at all times was expressed by several participants across all groups:

Use will be restricted cause most people do not have data on their phones nor will they spend money [on data] just to use the app. So it is good that you can do stuff that don’t need to connect. It’s a critical thing, for us.

Young adult focus group

##### Confirming the Final Functions and Features for Manaaki

At the conclusion of the second focus group session, participants confirmed that the core desired features and functions were clearly identified and reflected their insights and experiences. The constant across all groups was that Manaaki would appeal to and be helpful to people seeking help for gambling-related problems. This is illustrated by the following conversation in the Māori focus group:

Wow, this is really going to be great for people.

M1

Yeah, we are awesome aye!

M2

Yeah, I can’t wait to see it all done.

M1

I am gonna tell people about it, man.

M3

Ka pai, whānau [well done].

M4

Confirming the Final Design Features and Functions Against the Conceptual Framework

The final action undertaken by the research team was to appraise the functions, features, and attributes within the underpinning conceptual framework. Table 2 presents the process outcomes.

**Table 2:** Confirming the final attributes to be included in Manaaki with associated conceptual frameworks.

Attributes and conceptual framework	Use case examples	Final features and functions for Manaaki
Personalization—developing self-awareness and insight [50-52]	To be able to add my comments on things that are working well Put reminders in To encourage retention, create a longitudinal experience or history as it makes it difficult to stop interacting with the app and difficult to delete Show where I have come from so it is useful to me Can add other information that is important to me	Personal journal that auto-adds from modules plus can add information independently Shared forum feature that links others in the study (opt-in) Each module has a progress bar Videos of people like me within modules and in resources Use of other languages to help convey complex terms or concepts. Te Reo Māori and Pasifika languages used where relevant Use of avatars or ability to add own photo
Internal support progress—activating personal strengths and resources and enhancing belief for successful change [54,55]	To add or interact within the app and receive unanticipated rewards or appreciation that help reinforce behavior Having information in smaller sections and showing easy actions to promote a sense of capability and then skill development Messages that pop up that are positive and keep me motivated Real-time notification messages to reinforce activities and help to interpret or reinforce the activity	Informative and timely messages and notifications created Action prompts to complete a topic or module Instant feedback is given when an activity completed; that is, summary of what the outcomes of an activity are Assessments can be repeated with scores showing past and current outcomes and an interpretation to help understanding Able to post specific activities into a journal
External support—relapse prevention [56]	Can quickly find other help Resources provided	Webchat link to national gambling helpline Additional services section includes links to treatment services Links to external resources and tools (such as budgeting tools)
Literacy tools—activating personal strengths and resources and enhancing belief for successful change [54,55]	To have new or novel information presented through videos or notifications or links if needed to read more Attracting the attention of the user is about relevance and supports the usefulness of the app Supports how to make changes and maintain these	Resources and additional help and tools section Audio options to help when literacy is a challenge (only in English) Links to other resources such as budgeting and web-based gambling blocker tools Links to trusted resources Use of animations Use of the information icons to provide definitions or interpretation for some words
Navigation—literacy	Being able to see information in a way that is relevant to me and minimizes unhelpful and inappropriate comparisons Creates achievements and motivations to keep me engaged Can add other information that is important to me	Suggestions of where to next depending on outcomes A range of distraction activities that are relevant to intended users
Engaging—targeting thoughts and feelings and activating behaviors [53]	Has a good look and feel Looks engaging Tells a story that I can identify with	Use of New Zealand imagery Tui guide that provides an explanation of the purpose of each module Animations
Secure and simple	It needs to be secure and private Easy to download I do not want to be online all the time to use it	Registration and log-in simple with clear direction App size low and cater to the lowest operating system as possible The main elements within the app can be used offline

## Discussion

### Principal Findings

This paper describes in detail the development of Manaaki, a CBT mobile app intervention designed to support reduction in gambling-related harm. The blending of theory, domain expert input with end-user insights, and lived experience in a collaborative approach is effective for designing mHealth applications [31,36,57]. Creating a visual identity for the new Manaaki app, which resonates with end users, was an important step in establishing and reflecting a sense of trust, connection, and value with the overall intent and purpose of the intervention. To the best of our knowledge, this study is the first to adapt an evidence- and CBT-based program for people experiencing gambling problems into an app-delivered program using a cocreation process with end users.

The demand for mHealth tools that provide interventions (behavior change and cognitive therapy) and generalized support directly to individuals is growing [58-60]. The opportunity to provide interventions for population groups who are unable or unwilling to access more traditional forms is substantial [61,62].

The value of presenting best-practice approaches and evidence in understandable ways (posters, images, and Adobe XD) and cocreating with end users on how they can help translate it into the app’s look and feel was an important process for creating a sense of ownership and investment across all aspects of the content, features, function, and format of the final Manaaki app. The use of draft designs and real-time modifiable design flows enabled end-user participants to discuss and identify attributes to be included in the app that would resonate with them. Among the focus group sessions, the research team was able to explore the findings through a theoretical lens, convert the concepts into app designs, and then represent them in ways that were easily understood. Concepts such as personalization were presented using the app user’s name and included the creation of a personal avatar. The concept of engagement was reflected in background designs depicting a journey, animation of tools, use of the Tui, and audio features. Empowerment was demonstrated with progress bars, shared forums, self-assessment, and notifications.

Similarly, the content presented back to the participants was adapted and personalized to reflect within-app responses. To support health literacy, words such as fallacy and recovery continued to be used but with the information icon next to them to help participants understand and interpret these terms. In some cases, words and concepts were converted to Reo Māori (the Māori language).

The importance of positively framed and strength-based content was vital. The inclusion of audio files and information icons again elicited positive participant remarks about the app being relevant and appropriate to them. Similar findings are reported elsewhere [63]. The unanticipated prompts (notifications) that support actionable recommendations or app navigation suggestions are tools used in other domains and jurisdictions, such as marketing for supporting retention and motivation [64].

Research on addiction recovery has reported the importance of conveying a sense of social connectedness [65]. The end-user participants in this study also mentioned this, and the inclusion of features such as the webchat, forum, and visibility of services were seen as adding a layer of connection and support within the digital modality.

Adopting learning from the marketing discipline [66] and creating the naming and visual identity for Manaaki (ie, brand) that resonated with participants was essential in establishing and reflecting on them a sense of ownership, trust, connection, and value.

Finally, the attributes of information security, data use, accessibility (ie, available to older mobile phone operating systems), and offline capability were noted as part of the overall app usability discussions; however, they did not overshadow the key aim of the process.

### Limitations

Owing to the nature of the research methodology of focus groups, this study also comprises some biased and motivated responses as described in other research using focus groups [67,68]. Nonetheless, our participants were a subset of the target group that focused on the intervention app. The lived experiences of the participants flavored all discussions and decisions and were woven within the final application. Although it may not be possible to suggest that the process and outcome may not adequately represent the wider population of people who experience harm from gambling, it is more likely to resonate than an intervention that does not include any end users [69,70]. Adding a final step in which a small cohort of end users actively used the final app prototype before initiating the clinical trial was not possible in this study because of the overall project timeframe. This step can offer insights that initial design processes cannot anticipate and as such, provide an opportunity to refine the final product by applying active use insights before release, further enhancing the potential for effectiveness.

### Conclusions

The importance of domain expert input and evidence-based content combined with end-user insights and involvement should not be undervalued. Weaving these strands and following an iterative process, the research team, with the participants, produced an app that was intentionally tailored to the functional needs of individuals seeking to reduce gambling harm. This process gave the second phase of this study, a randomized waitlist control study to test the effectiveness of Manaaki—a CBT mHealth intervention—the best chance of demonstrating effectiveness. Although adopting this approach does not guarantee success, it is more likely to provide evidence of fidelity to the outcomes of the intervention. Manaaki is currently being trialed using a pragmatic randomized controlled trial with a waitlist control design [43]. This trial was completed in August 2021 and analyses are underway.

## Abbreviations

CBT: cognitive behavioral therapy
mHealth: mobile health
